# Prion Disease in Dromedary Camels, Algeria

**DOI:** 10.3201/eid2406.172007

**Published:** 2018-06

**Authors:** Baaissa Babelhadj, Michele Angelo Di Bari, Laura Pirisinu, Barbara Chiappini, Semir Bechir Suheil Gaouar, Geraldina Riccardi, Stefano Marcon, Umberto Agrimi, Romolo Nonno, Gabriele Vaccari

**Affiliations:** Ecole Normale Superieure Ouargla Laboratoire de Protection des Écosystèmes en Zones Arides et Semi Arides University Kasdi Merbah Ouargla, Ouargla, Algeria (B. Babelhadj);; Istituto Superiore di Sanità Department of Food Safety, Nutrition and Veterinary Public Health, Rome, Italy (M.A. Di Bari, L. Pirisinu, B. Chiappini, G. Riccardi, S. Marcon, U. Agrimi, R. Nonno, G. Vaccari);; Laboratoire de Physiopathologie et Biochimie de la Nutrition University Abou Bekr Bélkaid, Tlemcen, Algeria (S.B.S. Gaouar)

**Keywords:** Prion disease, camel prion disease, *Camelus dromedarius*, dromedary camels, prions, zoonoses, bovine spongiform encephalopathy, Creutzfeldt-Jakob disease

## Abstract

Prions cause fatal and transmissible neurodegenerative diseases, including Creutzfeldt-Jakob disease in humans, scrapie in small ruminants, and bovine spongiform encephalopathy (BSE). After the BSE epidemic, and the associated human infections, began in 1996 in the United Kingdom, general concerns have been raised about animal prions. We detected a prion disease in dromedary camels (*Camelus dromedarius*) in Algeria. Symptoms suggesting prion disease occurred in 3.1% of dromedaries brought for slaughter to the Ouargla abattoir in 2015–2016. We confirmed diagnosis by detecting pathognomonic neurodegeneration and disease-specific prion protein (PrP^Sc^) in brain tissues from 3 symptomatic animals. Prion detection in lymphoid tissues is suggestive of the infectious nature of the disease. PrP^Sc^ biochemical characterization showed differences with BSE and scrapie. Our identification of this prion disease in a geographically widespread livestock species requires urgent enforcement of surveillance and assessment of the potential risks to human and animal health.

Prions are responsible for a group of fatal and transmissible neurodegenerative diseases named prion diseases. A misfolded and aggregated isoform of a cell-surface protein termed cellular prion protein (PrP^Sc^) is the main, if not the sole, component of prions (*1*). Creutzfeldt-Jakob disease in humans and scrapie in small ruminants are the longest known diseases in this group, but prion diseases entered the public spotlight with the massive bovine spongiform encephalopathy (BSE) epidemic started in 1986 in the United Kingdom, revealing the zoonotic potential of animal prions.

Since the BSE epidemic begin, interest in these diseases has increased, and the prion universe has continued to expand (*2*). Several new prion diseases—including variant Creutzfeldt-Jakob disease, atypical/Nor98 scrapie of sheep, and atypical L- and H-type BSE—have been identified in the past 20 years, and chronic wasting disease (CWD) is spreading dramatically across cervid populations in North America and recently was discovered in Norway (*3*).

Public health concern increased markedly after variant Creutzfeldt-Jakob disease was demonstrated to be caused by the same prion strain responsible for the BSE epidemics (*4*). Unprecedented efforts were made to control the epidemics in cattle and to contain the exposure of humans to potentially infected cattle-derived materials.

In addition to having fatal consequences for infected animals, scrapie and BSE have a serious economic effect on the livestock industry. Scrapie brings economic damages through production loss, export loss, and increased cost for carcass disposal, which account for $10–$20 million annually in the United States (*5*). In the United Kingdom, where BSE was diagnosed in >180,000 cattle and up to 3 million were likely to have been affected, the cost to the public was >£5 billion (≈$7.1 billion US) (*6*).

Prion diseases can manifest as sporadic (putatively spontaneous), genetic, or infectious disorders (*1*). In animals, disorders resembling sporadic or genetic human prion diseases have been reported only recently, with the discovery of atypical/Nor98 scrapie in small ruminants (*7*) and L- and H-type BSE in cattle (*8**,**9*). Infectious prion diseases have been known for much longer and have been described in several animal species. Some diseases derived from accidental transmission, as is the case with BSE, which affected millions of cattle but also involved goats, domestic cats, nonhuman primates, and wild bovid and felid species, most likely fed with material contaminated by the BSE agent (*10*). Even the outbreaks of transmissible mink encephalopathy reported in the United States and various European countries in ranch-raised mink most likely originated from feedstuff accidentally contaminated by prions (*10*).

Despite the long list of susceptible animal species, prion diseases behave as infectious and naturally occurring conditions only in ruminants. Scrapie affects sheep and goats, and CWD affects different species of the *Cervidae* family: mule deer (*Odocoileus hemionus*), white-tailed deer (*O. virginianus*), elk (*Cervus canadensis*), and moose (*Alces alces*) (*11*). Furthermore, CWD has been recently diagnosed in reindeer (*Rangifer tarandus*) (*3*) and moose (*12*) from Norway.

We report prion disease in dromedary camels (*Camelus dromedarius*) from a Saharian population in Ouargla in southeastern Algeria, where the disease was observed in animals brought for slaughter at the Ouargla abattoir. Dromedaries are widespread throughout northern and eastern Africa, the Middle East, and part of Asia, where they are the means of subsistence for millions of families who live in the most hostile ecosystems on the planet. Since ancient times, camels have been exploited as beasts of burden and sources of milk and meat and for riding; today, they are tremendously important as a sustainable livestock species. During the past 10 years, the camel farming system has evolved rapidly and improved substantially (*13*). The emergence of a prion disease in a farmed animal species of such importance requires a thorough risk assessment for implementing evidence-based policies to control the disease in animals and minimize human exposure.

## Materials and Methods

### Animals and Tissue Samples

The Ouargla abattoir is one of the largest slaughterhouses in slaughtered volume for cattle, camels, and small ruminants in Algeria. In the past 5 years, neurologic symptoms have been observed more often in adult dromedaries at antemortem examination. The signs include weight loss; behavioral abnormalities; and neurologic signs, such as tremors, aggressiveness, hyperreactivity, typical down and upward movements of the head, hesitant and uncertain gait, ataxia of the hind limbs, occasional falls, and difficulty getting up (Video 1; Video 2).

**Video 1:**
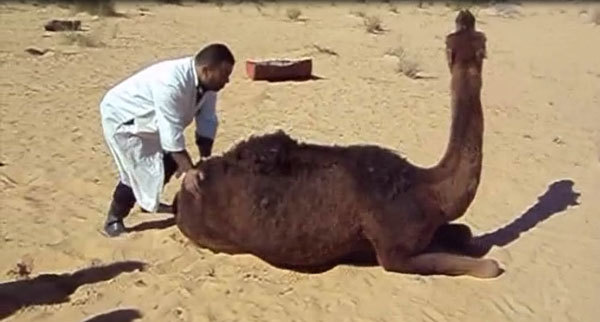
Dromedary camel found in the desert with difficulty getting up. At the abattoir, the animal showed aggressiveness (kicking). It became nervous when forced to cross an obstacle and showed the down and upwards movements of the head and teeth grinding. (Ahead of print - Video available in finalized issue)

**Video 2:**
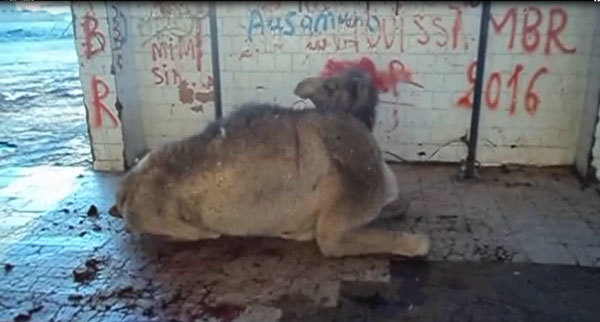
Dromedary camel waiting at the Ouargla abattoir for antemortem inspection. The animal shows down and upwards movements of the head and teeth grinding. (Ahead of print - Video available in finalized issue)

According to breeders’ descriptions, the early stage of the disease was mainly characterized by behavioral signs, such as loss of appetite and irritability. Separation from the herd at pastures along with aggressiveness and tendency to kick and bite when handled were usually observed. With disease progression, neurologic signs became obvious; animals showed ataxia that eventually led to recumbency and death.

Breeders reported that signs progressed slowly and that the duration of disease varied from 3 to 8 months. Although it has not been possible to date back the first cases of illness, information gathered from breeders and slaughterhouse personnel suggests the illness has been present since the 1980s.

Prion disease was suspected in dromedaries brought to the abattoir on the basis of clinical signs. 

We collected brain samples from 3 dromedaries (nos. 3, 4, and 8) showing neurologic symptoms and from 1 clinically healthy animal (no. 5), as well as cervical, prescapular, and lumbar aortic lymph nodes from 1 animal (no. 8). The animals were all females, belonging to the Sahraoui population, 10, 11, 13, and 14 years of age, respectively. 

We fixed samples in formalin for histologic and immunohistochemical examination. We also collected frozen brain samples from animals 4 and 8 for Western blot and genetic analysis and sampled formalin-fixed brain tissue from a clinically healthy animal (no. 5). We obtained brain samples from BSE-infected cattle and from ARQ/ARQ sheep, either naturally affected by scrapie or experimentally infected with BSE, from the surveillance system in Italy or from previous studies (*14*).

### Neuropathologic, Immunohistochemical, and Paraffin-Embedded Tissue Blot Analyses

We embedded brain and lymph node samples in paraffin wax, sectioned at 5 μm, and stained with hematoxylin and eosin or subjected to immunohistochemical or paraffin-embedded tissue blot analysis. We pretreated sections for immunohistochemistry with 98% formic acid for 5 min, followed by autoclaving in citrate buffer for 5 min at 121°C. We then treated sections with 6% normal goat serum (Vector Laboratories, Burlingame, CA, USA) in phosphate-buffered saline for 60 min. We performed immunohistochemical detection of PrP^Sc^ with L42 monoclonal antibody (mAb) (R-Biopharm, Darmstadt, Germany) at 0.01 μg/mL in phosphate-buffered saline overnight at 4°C. We treated sections with secondary biotinylated mouse antibody (Vector Laboratories), ABC Complex (Vector Laboratories) for 45 min, and diaminobenzidine (Sigma-Aldrich, St. Louis, MO, USA) for 3 min. We used Mayer’s hematoxylin for counterstaining. Each run comprised positive- and negative-control sections. We analyzed 3 sections from each lymph node sample.

We collected sections for paraffin-embedded blot on prewetted 0.45-μm–pore nitrocellulose membranes (Schleicher & Schuell, Dassel, Germany) and dried membranes for 24 h at 55°C. We performed membrane treatments, proteinase K (PK) (Sigma-Aldrich) digestion (50 μg/mL), and immunodetection as described (*15*). We used mAb L42 (0.01 μg/mL) as the primary antibody.

### Western Blot Analysis

We performed Western blot analysis of PrP^Sc^ from brain homogenates as previously described (*16*) and performed preliminary diagnosis with a final concentration of PK at 50 μg/mL. To compare dromedary PrP^Sc^ with PrP^Sc^ from sheep and cattle prion diseases, we performed molecular typing of their protease-resistant cores (PrP^res^) by discriminatory immunoblotting, conducted according to the ISS (Istituto Superiore di Sanità) discriminatory Western blot method (*17*) with minor modifications. The principle of discrimination is based on the differential N terminal cleavage by PK (200 μg/mL), revealed by using N terminal mAb with an epitope that is partially lost after PK digestion of BSE samples (*14**,**18*). As an additional discriminatory parameter, we measured the relative proportions of diglycosylated, monoglycosylated, and unglycosylated PrP fragments in L42 blots.

We performed deglycosylation by adding 18 μL of 0.2 M sodium phosphate buffer (pH 7.4) containing 0.8% Nonidet P40 (Roche. Penzberg, Germany) and 2 μL (80 U/mL) di N-Glycosidase F (Roche) to 5 μL of denaturated samples and incubating overnight at 37°C with gentle shaking. The mAbs used and their epitope on ovine PrP were as follows: L42 (148–153), 12B2 (93–97), SAF32 (octarepeat).

### PrP Gene Sequence Analysis

We extracted DNA from 100 mg of frozen brain tissue with DNeasy Blood and Tissue Kit (QIAGEN, Hilden, Germany) following the manufacturer’s instructions. We amplified the PrP gene (*PRNP*) coding sequence in a 50-μL final volume using 5 μL of extracted DNA, 1× AmpliTaq Gold 360 PCR Buffer (Applied Biosystems, Foster City, CA, USA), 2.5 mmol/L MgCl2, 1× 360 GC Enhancer, 200 μmol/L dNTPs, 0.25 μmol/L of forward (5′-GCTGACACCCTCTTTATTTTGCAG-3′) and reverse (5′-GATTAAGAAGATAATGAAAACAGGAAG-3′) primers (*19*), and 0.5 μL of AmpliTaq Gold 360 (Applied Biosystems), according to the following amplification protocol: 5 min at 96°C; 30 s at 96°C, 15 s at 57°C, 90 s at 72°C for 40 cycles, and 4 min at 72°C.

We purified amplicons by using an Illustra ExoProStar 1-Step clean-up kit (GE Healthcare Life Sciences, Little Chalfont, UK). We conducted sequencing reactions by using the BigDye Terminator v1.1 Cycle Sequencing Kit, purified using BigDye XTerminator Purification Kit, and detected with the ABI PRISM 3130 apparatus (all Applied Biosystems). We analyzed sequences by using Seq Scape version 2.5 (Applied Biosystems).

## Results

**Figure 1:**
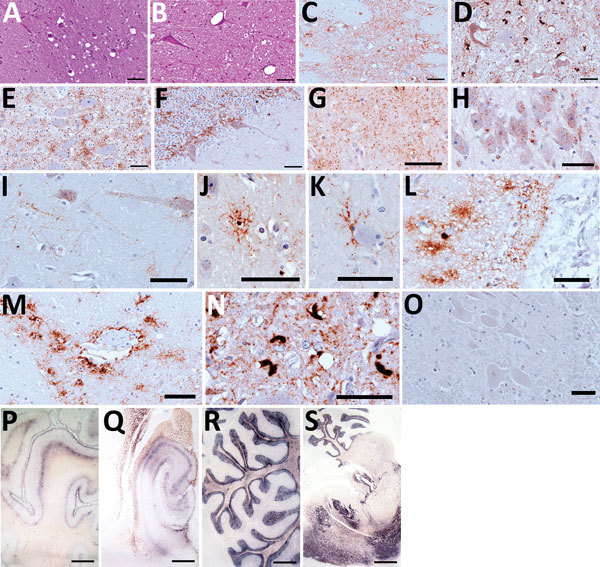
Hematoxylin and eosin staining (A, B), immunohistochemistry (C–O), and paraffin-embedded tissue blot analysis (P–S) of brains of dromedary camels brought for slaughter to the Ouargla abattoir, Algeria, 2016–2017. Spongiform change of neuropil, gliosis, and neuronal loss in thalamus (A) and intraneuronal vacuolation in pons (B) (scale bar = 50 μm). Immunohistochemistry for prion protein (PrP^Sc^) with L42 monoclonal antibody evidenced dense synaptic/punctate deposition in thalamus (C) and intraneuronal and extraneuronal PrP^Sc^ deposits in pons (D), accompanied by spongiform change. Perineuronal, diffused in neuropil and glial-associated PrP^Sc^ staining were also observed in the nucleus of the solitary tract (E) and cerebellum (F), which showed rare vacuoles (scale bars = 50 μm). Immunohistochemical analysis performed on brains of symptomatic dromedaries revealed several PrP^Sc^ deposition patterns, such as synaptic/punctate pattern diffused in the neuropil (G); intraneuronal deposition in pyramidal cells of hippocampus (H); perineuronal and linear staining in frontal cortex (I); intraglial PrP^Sc^ deposition (J–L); perivascular deposition (M); atypical intracellular PrP^Sc^ deposition pattern in pons (N). PrP^Sc^ was absent in asymptomatic dromedary used as negative control (O) (scale bars = 50 μm). PrP^Sc^ distribution, by paraffin-embedded tissue blot analysis, was observed in several brain areas, such as prefrontal cortex (P), hippocampus (Q), cerebellum (R), and a sagittal section of pons (S) (scale bar = 3 mm).

Histopathologic examination showed spongiform change, gliosis, and neuronal loss in several brain areas of the 3 symptomatic animals (Figure 1, panels A, B) but not in the asymptomatic dromedary. We observed vacuoles preferentially in the neuropil (Figure 1, panel A) but also frequently involving the neuronal bodies (Figure 1, panel B). Confluent vacuoles were rarely observed. These neurodegenerative changes consistently occurred in gray matter of subcortical brain areas, such as striatum, thalamus (Figure 1, panel A), midbrain, and pons (Figure 1, panel B) of all 3 animals; white matter was rarely affected. We observed moderate vacuolation in medulla oblongata, particularly in the vestibular and the olivary nucleus; nucleus of solitary tract and hypoglossal nucleus were less often affected. Cervical medulla, available only for animal 8, showed no spongiform changes. Cortical brain areas were variably involved. Animals 3 and 8 showed dispersed vacuolation in cingulate, piriform, and frontal cortices. In contrast, cerebral cortices were more heavily affected in animal 4. Cerebellum was collected from animals 4 and 8, and vacuoles were observed only in the molecular layer of animal 4.

By immunohistochemical analysis, we detected PrP^Sc^ in the brain of all symptomatic dromedaries. Overall, PrP^Sc^ deposition was invariably observed in brain areas with spongiform degeneration (Figure 1, panels C, D). In addition, PrP^Sc^ deposits also involved areas less often affected or not affected by spongiosis, such as the nucleus of the solitary tract (Figure 1, panel E); the hypoglossal nucleus; pyramidal cells of hippocampus; the granular layer of cerebellum, including Purkinje cells (Figure 1, panel F); and several white matter areas.

PrP^Sc^ deposition patterns involving neuropil, neurons, and glia differed. Patterns included synaptic/punctate (Figure 1, panel G), intraneuronal (Figure 1, panel H), perineuronal and linear (Figure 1, panel I), intraglial (Figure 1, panels J–L), and perivascular (Figure 1, panel M). In pons and medulla oblongata, we frequently observed an atypical intracellular pattern (Figure 1, panel N) in which PrP^Sc^ filled the whole cytoplasm. PrP^Sc^ was absent in the brain of the asymptomatic dromedary (Figure 1, panel O). Prominent protease-resistant PrP^Sc^ deposition was easily detected by paraffin-embedded blot in the same brain areas found positive by immunohistochemical analysis, such as the deep layers of cortices (Figure 1, panel P), the pyramidal layer and fimbria of hippocampus (Figure 1, panel Q), the granular layer of cerebellum and the associated white matter (Figure 1, panel R), and the gray matter of pons (Figure 1, panel S).

**Figure 2:**
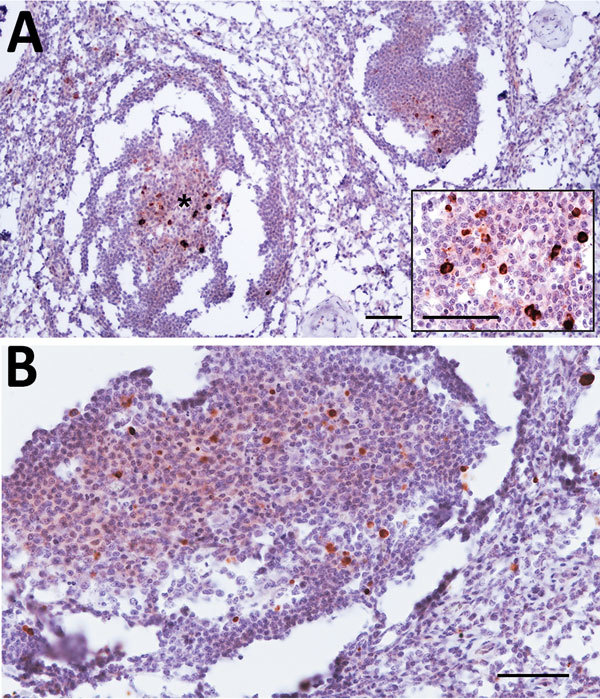
Prion protein immunolabeling in the germinal center of lymphoid follicles of cervical (A) and prescapular (B) lymph nodes of dromedary camel no. 8, Ouargla abattoir, Algeria. The architecture of lymph nodes appears moderately compromised by the partial freezing of samples that accidentally occurred before fixation. Scale bars = 50 μm. Inset in panel A: higher magnification showing the germinal center marked with asterisk; scale bar = 25 mm.

We detected PrP^Sc^ deposits in cervical, prescapular, and lumbar aortic lymph nodes from animal 8 (Figure 2) that involved >80% of primary and secondary follicles in the 3 sections analyzed. PrP^Sc^ deposits consisted of a reticular network at the center of the lymphoid follicles, which varied in staining intensity, accompanied by fine to coarse granules of PrP^Sc^ in the cytoplasm of nonlymphoid cells within the follicle. We also observed additional granular or intracellular PrP^Sc^ immunolabeling in the interfollicular areas.

**Figure 3:**
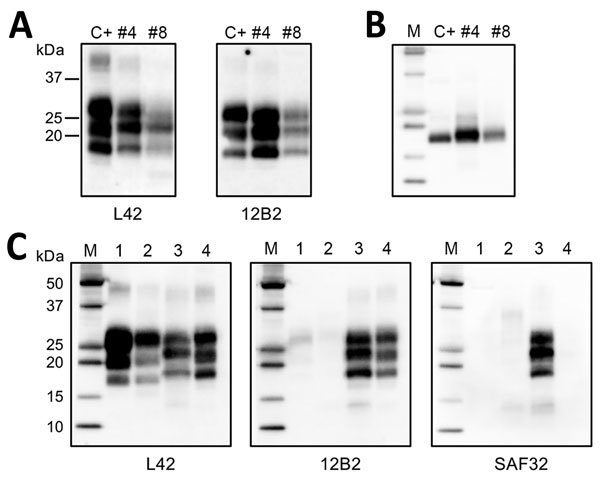
Western blot analysis of protein-resistant core (PrP^res^) of pathological dromedary prion protein. A) Western blot analysis of proteinase K (PK)–treated PrP^Sc^ in brain homogenates from dromedary camels with neurologic symptoms (nos. 4 and 8), Algeria. A sample of sheep scrapie was loaded as control (indicated as C+). Membranes were probed with L42 (left) and 12B2 monoclonal antibody (mAb) (right). Molecular weights (kDa) are indicated on the left. Tissue equivalents loaded per lane were 2 mg for camel samples and 0.1 mg for sheep scrapie. B) Samples after deglycosylation. Membrane was probed with L42 mAb. C) Comparison of dromedary PrP^res^ (from camel no. 4) with sheep bovine spongiform encephalopathy (BSE), bovine BSE, and sheep scrapie samples by ISS (Istituto Superiore di Sanità) discriminatory Western blot (*17*). Tissue equivalents loaded per lane were 2 mg for dromedary camel and bovine samples and 0.1 mg for sheep samples. In each blot, samples were loaded as follows: lane 1, ovine BSE; lane 2, bovine BSE; lane 3, dromedary camel no. 4; lane 4, ovine scrapie. Membranes were probed with L42, 12B2, and SAF32 mAbs, as indicated. For the analyses in panels B and C, protein standards were loaded and are indicated as M.

Western blot analysis of brain homogenates from dromedaries 4 and 8 revealed PrP^Sc^ with a PrP^res^ showing the classical electrophoretic profile, characterized by 3 main bands representing diglycosylated, monoglycosylated, and unglycosylated PrP^res^ (Figure 3, panel A). Accordingly, the 3 bands were resolved in a single band of ≈18 kDa after enzymatic deglycosylation (Figure 3, panel B).

The apparent molecular weight of PrP^res^ from both animals was slightly higher than classical scrapie and clearly higher than BSE and sheep passaged BSE (Figure 3, panels A, C, left side). This finding prompted us to investigate the N terminal PK cleavage under stringent PK conditions by discriminatory immunoblotting, which enables the molecular discrimination of the most common ruminant TSE strains from classical BSE (*14**,**18**,**20*). Epitope mapping of PrP^res^ showed that the higher apparent molecular weight in dromedary PrP^res^ reflects a more N terminal cleavage site than with BSEs and scrapie samples. Indeed, upon treatment with PK, dromedary PrP^res^ preserved the N terminal 12B2 and SAF32 mAb epitopes, whereas classical scrapie lost the SAF32 mAb epitope while preserving the 12B2 mAb epitope, and BSE samples lost both epitopes, being negative with SAF32 and 12B2 mAbs (Figure 3, panel C). We have previously shown that, with the ISS discriminatory Western blot, BSE and scrapie are both characterized by a diglycosylated dominant PrP^res^ pattern, although BSE is more heavily glycosylated than scrapie (*18*). Our data confirm this difference and show that PrP^res^ from dromedary camels is further less glycosylated than classical scrapie, being characterized by a monoglycosylated dominant PrP^res^ (Technical Appendix Figure). Sequencing revealed the same *PRNP* sequence in animals 4 and 8 (GenBank accession nos. MF990558–9), which, in turn, showed 100% nt identity with the *PRNP* sequence already reported for dromedary camels (*19*).

In parallel to the laboratory analyses, we undertook a retrospective investigation of neurologic signs in dromedaries at the Ouargla slaughterhouse. Twenty of 937 animals in 2015 and 51 of 1,322 in 2016 showed the previously described neurologic signs (Table); the overall prevalence was 3.1% in dromedaries brought for slaughter. All slaughtered animals derived from the area surrounding Ouargla, and the disease was observed only in animals >8 years of age.

**Table:** Suspected prion disease in dromedary camels at antemortem inspection at the Ouargla slaughterhouse, Algeria

Month	2015			2016	
	No. animals presented at abattoir	No. with clinically suspected prion disease		No. animals presented at abattoir	No. with clinically suspected prion disease
Jan	63	0		67	3
Feb	70	2		83	4
Mar	86	1		73	3
Apr	79	2		85	3
May	97	3		93	4
Jun	81	1		117	5
Jul	92	2		135	6
Aug	121	4		145	7
Sep	31	1		44	5
Oct	42	1		110	4
Nov	89	2		164	4
Dec	86	1		206	3
Total	937	20		1,322	51

## Discussion

We describe a prion disease in dromedary camels, designated as camel prion disease (CPD), that we detected during routine antemortem inspection at the Ouargla slaughterhouse in Algeria. Retrospective analysis indicated a 3.1% prevalence of animals with neurologic signs suggestive of the disease in dromedaries brought for slaughter. That figure appears to be reliable given that clinical suspicion was confirmed in all 3 animals undergoing laboratory analysis. However, because prion diseases are characterized by long incubation periods and the age at which the disease becomes apparent (>8 years) is more advanced than the age at which most dromedaries are slaughtered (<5 years), the prevalence found in the older animals is probably higher than the actual prevalence (excluding younger animals). 

The spectrum of animal species susceptible to prion disease is large. However, only in ruminants belonging to the *Bovidae* and *Cervidae* families do prion diseases behave as infectious and naturally occurring conditions. Dromedaries are not ruminants (suborder *Ruminantia*) but rather are *Tylopoda*, a suborder of *Artiodactyla*, which also includes the 2-humped camel (*Camelus bactrianus*), wild Bactrian camel (*C. ferus*), llamas (*Lama glama*), alpacas (*Vicugna pacos*), and vicuñas (*V. vicugna*) (*21*). The presence of a prion disease in dromedaries extends the spectrum of animal species naturally susceptible to prion diseases to taxa different from those already known and opens up new research areas on the ecology and the host–pathogen relationship of prion diseases.

Whether CPD is an infectious disease in natural conditions is a key question. In scrapie and CWD, in which lymphoid tissues are extensively involved, the horizontal transmission in natural conditions is efficient. In contrast, when the peripheral lymphoid tissues are not substantially involved, as in cattle BSE, atypical/Nor98 scrapie, and most human prion diseases, the horizontal transmission appears to be inefficient. This inefficiency usually is explained by assuming the in vivo dissemination of PrP^Sc^ to the periphery as a prerequisite to facilitate prion shedding into the environment (*22*). Although we obtained samples from a single animal, our detection of PrP^Sc^ in all lymph nodes available suggests an abundant extraneural pathogenesis and, along with the notable prevalence of clinical cases at the slaughterhouse, concurs to suggest the infectious nature of CPD. These observations also suggest that the disease has an acquired rather than spontaneous onset.

The origin of CPD is unknown. It might be a disease unique to dromedaries or a malady deriving from transmission of a prion disease from another species. It is worth noting that meat and bone meal has been exported from the United Kingdom worldwide, and after the ban on feeding animals with ruminant protein in 1988, export to the Third World had soared to 30,000 tons (*23*) in 1991. Thus, the possibility that BSE-infected feed could have reached North Africa cannot be ruled out. However, even if the risk for BSE has not been formally assessed in Algeria and an official surveillance system for animal prion diseases is lacking, BSE is unlikely to appear in dromedaries without evidence in cattle populations. Moreover, dromedaries are mostly raised with no use of feedstuff. Lastly, the PrP^Sc^ biochemical signature in CPD clearly differs from that of BSE or sheep-passaged BSE. Although host factors are known to be able to alter the PrP^Sc^ signature during interspecies transmission, the BSE profile generally has been preserved in species accidentally or experimentally affected. In principle, CPD also might have derived from scrapie. Dromedaries often are raised along with sheep and goats, sharing common pastures. However, although the absence of an effective surveillance system prevents drawing any conclusions, scrapie has never been reported in Algeria, and a field survey in northeastern Algeria could not provide evidence of the disease (*24*). Moreover, the PrP^Sc^ signature of CPD differed from the classical scrapie case used for comparison (Figure 3). To help clarify the origin and nature of CPD, bioassays in a panel of rodent models are ongoing for a thorough prion strain characterization.

Future investigations of the geographic distribution of CPD will help clarify its origin. If the disease is confined to the dromedary populations of the Ouargla region, a localized event of transmission could be hypothesized. Common-source scrapie epidemics in sheep and goats occurred in the United Kingdom and Italy as a consequence of the use of accidentally contaminated vaccines (*25**,**26*). However, in the Ouargla region, no vaccination program has been implemented for infectious disease prophylaxis in dromedaries. Intriguingly, dromedary breeders indicate that the only food source other than pasture available to dromedaries in the Ouargla region are the waste dumps widespread in the desert near the oil extraction plants, where dromedaries and small ruminants gather and scavenge (Video 3). The possibility that dromedaries acquired the disease from eating prion-contaminated waste needs to be considered.

**Video 3:**
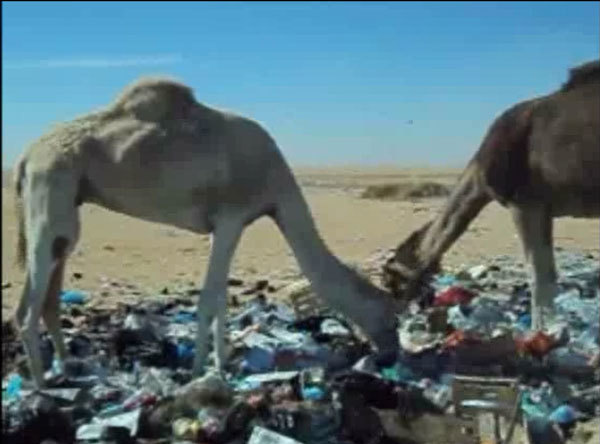
Dromedary camels gathering and scavenging the waste dumps in the desert near an oil extraction plant. (Ahead of print - Video available in finalized issue)

Tracing the origin of prion diseases is challenging. In the case of CPD, the traditional extensive and nomadic herding practices of dromedaries represent a formidable factor for accelerating the spread of the disease at long distances, making the path of its diffusion difficult to determine. Finally, the major import flows of live animals to Algeria from Niger, Mali, and Mauritania (*27*) should be investigated to trace the possible origin of CPD from other countries.

Camels are a vital animal species for millions of persons globally. The world camel population has a yearly growth rate of 2.1% (*28*). In 2014, the population was estimated at ≈28 million animals, but this number is probably underestimated. Approximately 88% of camels are found in Africa, especially eastern Africa, and 12% are found in Asia. Official data reported 350,000 dromedaries in Algeria in 2014 (*28*).

On the basis of phenotypic traits and sociogeographic criteria, several dromedary populations have been suggested to exist in Algeria (*29*). However, recent genetic studies in Algeria and Egypt point to a weak differentiation of the dromedary population as a consequence of historical use as a cross-continental beast of burden along trans-Saharan caravan routes, coupled with traditional extensive/nomadic herding practices (*30*).

Such genetic homogeneity also might be reflected in *PRNP*. Studies on *PRNP* variability in camels are therefore warranted to explore the existence of genotypes resistant to CPD, which could represent an important tool for CPD management as it was for breeding programs for scrapie eradication in sheep.

In the past 10 years, the camel farming system has changed rapidly, with increasing setup of periurban dairy farms and dairy plants and diversification of camel products and market penetration (*13*). This evolution requires improved health standards for infectious diseases and, in light of CPD, for prion diseases.

The emergence of another prion disease in an animal species of crucial importance for millions of persons worldwide makes it necessary to assess the risk for humans and develop evidence-based policies to control and limit the spread of the disease in animals and minimize human exposure. The implementation of a surveillance system for prion diseases would be a first step to enable disease control and minimize human and animal exposure. Finally, the diagnostic capacity of prion diseases needs to be improved in all countries in Africa where dromedaries are part of the domestic livestock.

Technical Appendix Relative proportions of diglycosylated, monoglycosylated, and unglycosylated bands in prion protein from sheep scrapie, sheep and bovine bovine spongiform encephalopathy, and dromedary camel samples.

## References

[1] Prusiner SB. Prions. Proc Natl Acad Sci U S A. 1998;95:13363–83. 10.1073/pnas.95.23.13363 9811807 PMC33918

[2] Watts JC, Balachandran A, Westaway D. The expanding universe of prion diseases. PLoS Pathog. 2006;2:e26. 10.1371/journal.ppat.0020026 16609731 PMC1434791

[3] Benestad SL, Mitchell G, Simmons M, Ytrehus B, Vikøren T. First case of chronic wasting disease in Europe in a Norwegian free-ranging reindeer. Vet Res (Faisalabad). 2016;47:88. 10.1186/s13567-016-0375-4 27641251 PMC5024462

[4] Bruce ME, Will RG, Ironside JW, McConnell I, Drummond D, Suttie A, et al. Transmissions to mice indicate that ‘new variant’ CJD is caused by the BSE agent. Nature. 1997;389:498–501. 10.1038/39057 9333239

[5] Greenlee JJ, Greenlee MH. The transmissible spongiform encephalopathies of livestock. ILAR J. 2015;56:7–25. 10.1093/ilar/ilv008 25991695

[6] Donnelly CA, Ferguson NM, Ghani AC, Anderson RM. Implications of BSE infection screening data for the scale of the British BSE epidemic and current European infection levels. Proc Biol Sci. 2002;269:2179–90. 10.1098/rspb.2002.2156 12427310 PMC1691156

[7] Benestad SL, Sarradin P, Thu B, Schönheit J, Tranulis MA, Bratberg B. Cases of scrapie with unusual features in Norway and designation of a new type, Nor98. Vet Rec. 2003;153:202–8. 10.1136/vr.153.7.202 12956297

[8] Casalone C, Zanusso G, Acutis P, Ferrari S, Capucci L, Tagliavini F, et al. Identification of a second bovine amyloidotic spongiform encephalopathy: molecular similarities with sporadic Creutzfeldt-Jakob disease. Proc Natl Acad Sci U S A. 2004;101:3065–70. 10.1073/pnas.0305777101 14970340 PMC365745

[9] Biacabe AG, Laplanche JL, Ryder S, Baron T. Distinct molecular phenotypes in bovine prion diseases. EMBO Rep. 2004;5:110–5. 10.1038/sj.embor.7400054 14710195 PMC1298965

[10] Sigurdson CJ, Miller MW. Other animal prion diseases. Br Med Bull. 2003;66:199–212. 10.1093/bmb/66.1.199 14522860

[11] Agrimi U, Nonno R, Dell’Omo G, Di Bari MA, Conte M, Chiappini B, et al. Prion protein amino acid determinants of differential susceptibility and molecular feature of prion strains in mice and voles. PLoS Pathog. 2008;4:e1000113. 10.1371/journal.ppat.1000113 18654630 PMC2453331

[12] Norwegian Scientific Committee for Food and Environment. CWD in Norway. Opinion of the panel on biological hazards. Oslo (Norway): Norwegian Scientific Committee for Food and Environment; 2016.

[13] Faye B, Jaouad M, Bhrawi K, Senoussi A, Bengoumi M. Elevage camelin en Afrique du Nord: état des lieux et perspectives. Revue d’élevage et de médecine vétérinaire des pays tropicaux. Rev Elev Med Vet Pays Trop. 2014;67:213–21. 10.19182/remvt.20563

[14] Migliore S, Esposito E, Pirisinu L, Marcon S, Di Bari M, D’Agostino C, et al. Effect of PrP genotype and route of inoculation on the ability of discriminatory Western blot to distinguish scrapie from sheep bovine spongiform encephalopathy. J Gen Virol. 2012;93:450–5. 10.1099/vir.0.035469-0 21994325

[15] Di Bari MA, Nonno R, Castilla J, D’Agostino C, Pirisinu L, Riccardi G, et al. Chronic wasting disease in bank voles: characterisation of the shortest incubation time model for prion diseases. PLoS Pathog. 2013;9:e1003219. 10.1371/journal.ppat.1003219 23505374 PMC3591354

[16] Pirisinu L, Marcon S, Di Bari MA, D’Agostino C, Agrimi U, Nonno R. Biochemical characterization of prion strains in bank voles. Pathogens. 2013;2:446–56. 10.3390/pathogens2030446 25437201 PMC4235696

[17] Community Reference Laboratory of the European Union. TSE strain characterization in small ruminants—a technical handbook for national reference laboratories in the EU. Version 8. December 2016 [cited 2017 Nov 10]. https://science.vla.gov.uk/tse-lab-net/documents/tse-oie-rl-handbook.pdf

[18] Pirisinu L, Migliore S, Di Bari MA, Esposito E, Baron T, D’Agostino C, et al. Molecular discrimination of sheep bovine spongiform encephalopathy from scrapie. Emerg Infect Dis. 2011;17:695–8. 10.3201/eid1704.101215 21470463 PMC3377410

[19] Kaluz S, Kaluzova M, Flint AP. Sequencing analysis of prion genes from red deer and camel. Gene. 1997;199:283–6. 10.1016/S0378-1119(97)00382-X 9358067

[20] Mazza M, Iulini B, Vaccari G, Acutis PL, Martucci F, Esposito E, et al. Co-existence of classical scrapie and Nor98 in a sheep from an Italian outbreak. Res Vet Sci. 2010;88:478–85. 10.1016/j.rvsc.2009.11.015 20031179

[21] Spaulding M, O’Leary MA, Gatesy J. Relationships of Cetacea (Artiodactyla) among mammals: increased taxon sampling alters interpretations of key fossils and character evolution. PLoS One. 2009;4:e7062. 10.1371/journal.pone.0007062 19774069 PMC2740860

[22] Gough KC, Maddison BC. Prion transmission: prion excretion and occurrence in the environment. Prion. 2010;4:275–82. 10.4161/pri.4.4.13678 20948292 PMC3268960

[23] Phillips NL, Bridgeman J, Ferguson-Smith MA. The BSE inquiry: return to an order of the Honourable the House of Commons dated October 2000 for the report, evidence and supporting papers of the inquiry into the emergence and identification of bovine spongiform encephalopathy (BSE) and variant Creutzfeldt-Jakob disease (vCJD) and the action taken in response to it up to 20 March 1996. London (UK): Stationery Office; 2000.

[24] Mohammed K, Yahia A, Abdelkader AA, Semir Bechir Suheil G. Epidemiological study of scrapie disease in local sheep population in Algeria. Genetics and Biodiversity Journal. 2017;1:26–9.

[25] Gordon WS. Advances in veterinary research. Vet Rec. 1946;58:516–25. 20279638

[26] Agrimi U, Ru G, Cardone F, Pocchiari M, Caramelli M. Epidemic of transmissible spongiform encephalopathy in sheep and goats in Italy. Lancet. 1999;353:560–1. 10.1016/S0140-6736(98)04545-0 10028993

[27] Kadim IT, Mahgoub O. ‎ Faye B,‎ Farouk MM, editors. Camel meat and meat products. Wallingford (UK): CABI; 2013.

[28] Food and Agriculture Organization of the United Nations. Live animals [cited 2017 Nov 10]. http://www.fao.org/faostat/en/#data/QA

[29] Amine CY, Samir GSB, Nasreddine M, Nacera TA, Nadhira S-M. Study of camelina biodiversity in southwestern of Algeria. J Life Sci. 2013;7:416.

[30] Cherifi YA, Gaouar SB, Guastamacchia R, El-Bahrawy KA, Abushady AM, Sharaf AA, et al. Weak genetic structure in northern African dromedary camels reflects their unique evolutionary history. PLoS One. 2017;12:e0168672. 10.1371/journal.pone.0168672 28103238 PMC5245891

