# Supplementary material for: Prion Disease in Dromedary Camels, Algeria
Source: Emerg Infect Dis. 2018 Jun;24(6):1029–36. doi: 10.3201/eid2406.172007 (PMC6004840; doi:10.3201/eid2406.172007)
Supplement: Technical Appendix — Relative proportions of diglycosylated, monoglycosylated, and unglycosylated bands in prion protein from sheep scrapie, sheep and bovine bovine spongiform encephalopathy, and dromedary camel samples. [file 17-2007-Techapp-s1.pdf]

Article DOI: https://doi.org/10.3201/eid2405.172007

Prion Disease in Dromedary Camels, Algeria

Technical Appendix

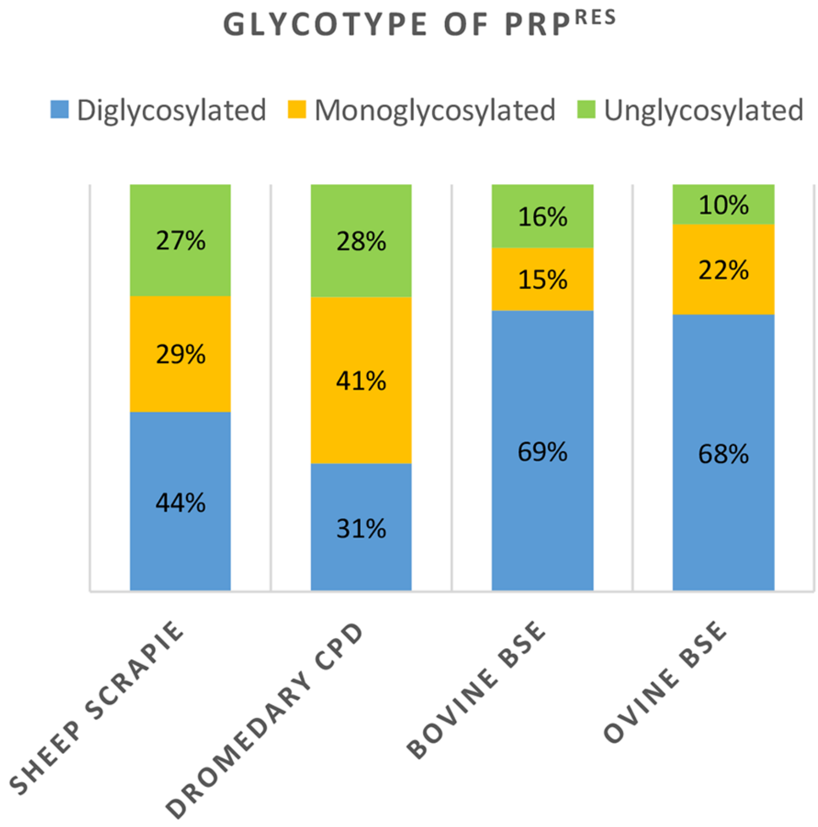

Technical Appendix Figure. Relative proportions of diglycosylated, monoglycosylated, and unglycosylated bands (“glycotype”) in prion protein (PrPres) from sheep scrapie, sheep bovine spongiform encephalopathy (BSE), bovine BSE, and dromedary camel samples, as measured by ISS Discriminatory Western blot with L42 monoclonal antibody.
